# Is there association between *Glutathione S Transferases* polymorphisms and cataract risk: a meta-analysis?

**DOI:** 10.1186/s12886-015-0065-4

**Published:** 2015-07-26

**Authors:** Wen Sun, Liling Su, Yan Sheng, Ye Shen, Guangdi Chen

**Affiliations:** Department of Ophthalmology, The First Affiliated Hospital, Zhejiang University School of Medicine, 79 Qingchun Road, Hangzhou, 310003 China; Department of Public Health, Zhejiang University School of Medicine, 866 Yuhangtang Road, 310058 Hangzhou, China

**Keywords:** *Glutathione S Transferases*, Polymorphisms, Cataract, Meta-analysis

## Abstract

**Background:**

*Glutathione S transferase* (*GST*) polymorphisms have been considered as risk factors for age-related cataracts, but the results remain controversial. In this study, we have performed a meta-analysis to evaluate the association between polymorphisms of *GSTM1* and *GSTT1* and cataract risk.

**Methods:**

Published literature from PubMed and other databases were retrieved. The case–control studies regarding the association between *GSTM1* or *GSTT1* polymorphism and cataract risk were included. Pooled odds ratio (OR) and 95 % confidence interval (CI) were calculated using random- or fixed-effects model.

**Results:**

Fifteen studies on *GSTM1* (3,065 patients and 2,105 controls), and nine studies on *GSTT1* (2,374 patients and 1,544 controls) were included. By pooling all the studies, *GSTM1* null polymorphism was not associated with cataract risk, and this negative association maintained in subgroup analyses. However, *GSTT1* null polymorphism was significantly associated with increased risk of posterior subcapsular (OR, 1.42; 95 % CI, 1.04–1.94) but not other subtypes of cataract. Stratified analyses demonstrated an association of *GSTT1* null genotype with increased risk of cataract in Asian (OR, 1.44; 95 % CI, 1.14–1.83) but not Caucasian populations. In addition, seven pooled studies showed no association of cataract risk with the combined *GSTM1* and *GSTT1* null genotypes.

**Conclusions:**

This meta-analysis suggests that *GSTT1* null polymorphism is associated with increased risk of posterior subcapsular cataract. Given the limited sample size, the association between *GSTT1* null polymorphism and cataract risk in Asian awaits further investigation.

## Background

Cataract is the opacification of eye lens with the breakdown of the lens protein microarchitecture, which adversely affects the transmission of light onto the retina [1]. Recent data suggest that cataract remains the leading cause of blindness worldwide, and the age-related cataract accounts for approximately 50 % of blindness cases [2]. Epidemiologic studies have revealed some environmental risk factors for age-related cataract, including ultraviolet B light exposure, ionizing radiation, smoking, and use of steroids [3]. Recently, genetic factors have been found to play important roles in the pathogenesis of age-related cataract [4]; furthermore, gene polymorphisms have been reported to be associated with age-related cataract risk [5, 6].

It has been reported that oxidative stress contributes to development of age-related cataract [7]. Biochemical evidence demonstrates that generation of excessive reactive oxygen species (ROS) results in abnormal degradation, cross linking, and aggregation of lens proteins, and is involved in cataractogenesis [8]. The oxidative damage during cataractogenesis can be alleviated by cellular defense mechanisms, including catalase, superoxide dismutase, glutathione peroxidase, and glutathione S transferases (GSTs) in the eye [9]. Among them, GSTs are a superfamily of enzymes that play important roles in the detoxification, elimination of xenobiotics and antioxidation, such as carcinogens, toxins, oxidants and drugs [10]. This enzymatic superfamily is composed of three different families: mitochondrial, microsomal and cytosolic. The cytosolic family of *GSTs* are classified in seven classes based on chromosomal location and on sequence similarity: alpha (GSTA), mu (GSTM), pi (GSTP), theta (GSTT), kappa (GSTK), zeta (GSTZ) and omega (GSTO) [11].

Previous studies have identified numerous variants in *GST* genes, and some of these polymorphisms are functional, e.g., *GSTT1* and *GSTM1* null polymorphisms [12]. In fact, the deletion of *GSTT1* or *GSTM1* results in dysfunction of their enzyme activity [12], and these polymorphisms of *GST* are associated with increased risks of various pathologies including cancers [13] and ophthalmologic problems such as glaucoma [14]. The relationships between *GST* polymorphisms and risks of age-related cataract have been studied for many years, and an early meta-analysis suggested that *GSTM1* and *GSTT1* null genotypes were associated with increased risk for senile cataract in Asians but not Caucasians [6]. However, recent studies showed that *GSTM1* positive (*GSTM1*^*+/+*^) genotype was associated with a susceptibility to age-related cortical cataract in Asians [15], while *GSTM1* or *GSTT1* null genotype was associated with age-related cataract risk in Caucasians [16, 17]. These inconsistent results may be due to the relatively small size of study populations from each individual study, or limited studies included by the previous meta-analysis; therefore, in this study we have conducted an update meta-analysis to reevaluate the associations between *GSTM1* and *GSTT1* polymorphisms and age-related cataract risk.

## Methods

### Identification of eligible studies

To identify all articles that evaluated the association of *GST* polymorphism with cataract, we carried out a literature search in the PubMed databases up to December 2014 with the following MeSH terms and keywords: “cataract”, “glutathione S transferase”, and “polymorphism”. The manual search was conducted to identify additional studies from other sources (e.g., Embase, Web of Knowledge, China National Knowledge Infrastructure), review articles on this topic or references to original studies. The inclusion criteria for eligible studies included in this meta-analysis as follows: (a) a study evaluating the association between *GSTM1* or *GSTT1* null polymorphism and cataract, (b) a case–control study, (c) an unrelated study, if studies had partly overlapped subjects, only the one with a larger sample size was selected, (d) a study with available genotype frequency, and (e) a study with sufficient data for estimating odds ratio (OR) and 95 % confidence interval (CI). Our meta-analysis was in accordance with PRISMA guidelines

Because the data included in this study were retrieved from the literatures, written informed consent for participation and ethical approval have been provided by original studies. Thus, all investigations analyzed in this meta-analysis have been carried out in compliance with the Helsinki Declaration.

### Data extraction

Two investigators (W.S. and L.S) independently assessed the articles for inclusion, and reached a consensus on data extracted. For each study, the following information was extracted: the first author name and publication year of the article; ethnicity (country) of study subjects; gene polymorphisms and genotype frequencies; sample size (numbers of cases and controls); sources of controls; subtypes of cataract classified. The missing data and information of included studies were obtained by contacting the study authors through email.

### Statistical analysis

The association between *GSTM1*, or *GSTT1* polymorphism and cataract was estimated by calculating pooled OR and 95 % CI. The significance of the pooled OR was determined by Z test, in which the *P* < 0.05 was considered statistically significant. The risk of *GSTM1* or *GSTT1* null genotype on cataract was evaluated by comparing to wild type homozygote as their reference. Stratified analyses were also performed by ethnicity of study populations, the source of controls, gender of subjects, and cataract subtype. Considering the possible additive effect of different *GST* genotypes, we next evaluated the association between the genotype profile and cataract risk, in which the individuals with two putative low-risk genotypes, i.e.,, the presence of functional *GSTM1* and *GSTT1* alleles, were used as reference group [18]. For the quantitative synthesis analysis, the environmental effects were not adjusted due to the lack of information from the original study. The I^2^-based Q statistic test was applied to examine variations due to heterogeneity rather than chance. A random-effects (DerSimonian-Laird method) model or fixed-effects (Mantel-Haenszel method) model was applied to calculate pooled effect estimates in the presence (*P* ≤ 0.10) or absence (*P* > 0.10) of heterogeneity. The Egger’s test [19] and the Begg’s [20] test were applied to detect publication bias for the overall pooled analysis of *GSTM1* or *GSTT1* null genotypes. Additionally, the Begg’s funnel plot was obtained, in which an asymmetry of the funnel plot indicates a potential publication bias. The one-way sensitivity analysis was performed when one single study was excluded each time, and the new pooled results reflect the influence of the study deleted to the overall OR. All analyses were carried out with Stata software (version 11.0; Stata Corp LP, College Station, TX), and the two-sided *P* values were applied.

## Results

### Characteristics of studies

**Fig. 1:**
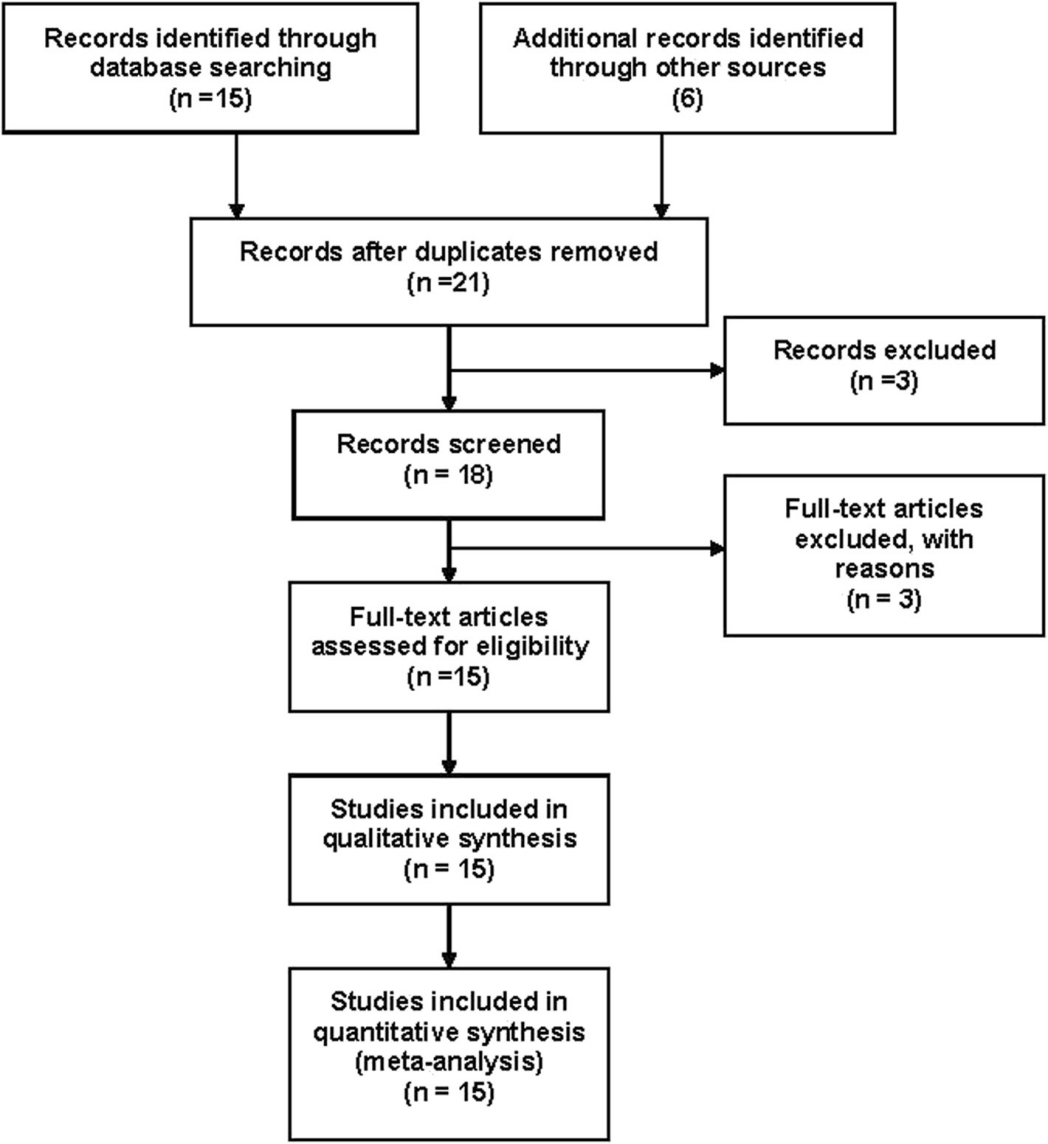
Flow diagram of studies identification

By searching PubMed, fifteen abstracts were retrieved through the search “cataract” “*glutathione S transferase*” and “polymorphism”, and nine studies meeting the inclusion criteria were identified as eligible [15–18, 21–25]. Out of the fifteen, one was meta-analysis [6] and one was laboratory study [26]. One article was excluded due to investigation on an association of presenile cataracts with heterozygosity for galactosaemic states and with riboflavin deficiency [27]. We excluded two articles on the relationship between *GST* polymorphisms and risk of age-related macular degeneration [28] or primary open-angle glaucoma [29]. We also excluded one article that examined the association of *GSTO* polymorphisms with cataract risk [30]. In addition, we included six eligible articles with manual searching [31–36]. As a result, a total of fifteen articles on *GSTM1* or *GSTT1* polymorphisms meeting the inclusion criteria were identified as eligible studies (Fig. 1).

Fifteen studies on *GSTM1* (3,065 cases and 2,105 controls), and nine studies on *GSTT1* (2,374 cases and 1,544 controls) were included in this meta-analysis. For the ethnicities, six studies of Asians and eight studies of Caucasians were included on the *GSTM1* genotype. As to *GSTT1*, two studies of Asians and six studies of Caucasians were included. We also grouped studies with different sources of controls (i.e., population-based or hospital-based), gender (male or female) and subtypes of cataracts (e.g., cortical, nuclear, posterior sub-capsular or mixed cataract). In addition to the study by Juronen *et al*. [25] that determined the *GSTM1* and *GSTT1* phenotypes by enzyme-linked immunosorbent assay (ELISA), the genotyping for *GSTM1*, or *GSTT1* was determined by polymerase chain reaction (PCR) assay in all other studies. The Table 1 presents the detailed characteristics of each study included in the meta-analysis.

**Table 1:** Characteristics of literatures included in the meta-analysis

Author/ Year	Country	Ethnicity	Sample size Cases/controls^a^	Source of controls	Cataract subtype
*GSTM1*					
Sekine 1995 [36]	Japan	Asian	138/62 (101/30)	PB	Not classified
Alberti 1996 [35]	United States	Caucasian	202/98 (99/49)	HB	NC/CC/M
Pi 1996 [34]	China	Asian	59/112 (41/57)	HB	Not classified
Hao 1999 [33]	China	Asian	77/76 (41/35)	HB	Not classified
Juronen 2000 [25]	Estonia	Caucasian	503/202 (240/111)	HB	CC/NC/ PSC/M
Saadat 2004 [24]	Iran	Caucasian	150/150 (90/58)	HB	Not classified
Saadat 2006 [23]	Iran	Caucasian	95/95 (56/36)	HB	Not classified
Guven 2007 [18]	Turkey	Caucasian	195/136 (105/58)	HB	CC/NC/ PSC/MC
Xu 2007 [32]	China	Asian	120/118 (81/60)	HB	Not classified
Azeem 2009 [22]	Egypt	Caucasian	53/73 (23/46)	HB	Not classified
Zhou 2010 [21]	China	Asian	279/145 (171/95)	PB	Not classified
Sireesha 2012 [16]	India	Caucasian	455/205 (177/94)	PB	CC/NC/ PSC/MC
Saadat 2012 [17]	Iran	Caucasian	186/195 (104/89)	HB	Not classified
Jiang 2012 [15]	China	Asian	422/312 (176/173)	HB	CC
Chandra 2014 [31]	India	Caucasian	124/126 (43/68)	HB	Not classified
*GSTT1*					
Juronen 2000 ^[ 25]^	Estonia	Caucasian	503/202 (73/36)	HB	CC/NC/PSC/MC
Saadat 2004 [24]	Iran	Caucasian	150/150 (49/46)	HB	Not classified
Guven 2007 [18]	Turkey	Caucasian	195/136 (29/22)	HB	CC/NC/PSC/MC
Azeem 2009 [22]	Egypt	Caucasian	53/73 (16/21)	HB	Not classified
Zhou 2010 [21]	China	Asian	279/145 (146/60)	PB	CC/NC/PSC
Sireesha 2012 [16]	India	Caucasian	455/205 (123/40)	PB	CC/NC/PSC/MC
Saadat 2012 [17]	Iran	Caucasian	186/195 (49/57)	HB	Not classified
Jiang 2012 [15]	China	Asian	422/312 (221/138)	HB	CC
Chandra 2014 ^[ 31]^	India	Caucasian	131/126 (18/5)	HB	Not classified

Abbreviations: *PB* population-based, *HB* hospital-based, *CC* cortical cataract, *NC* nuclear cataract, *PSC* posterior sub-capsular cataract, *MC* mixed cataract

^a^The number of null genotype cases or controls was presented in parenthesis

### Quantitative synthesis

**Fig. 2:**
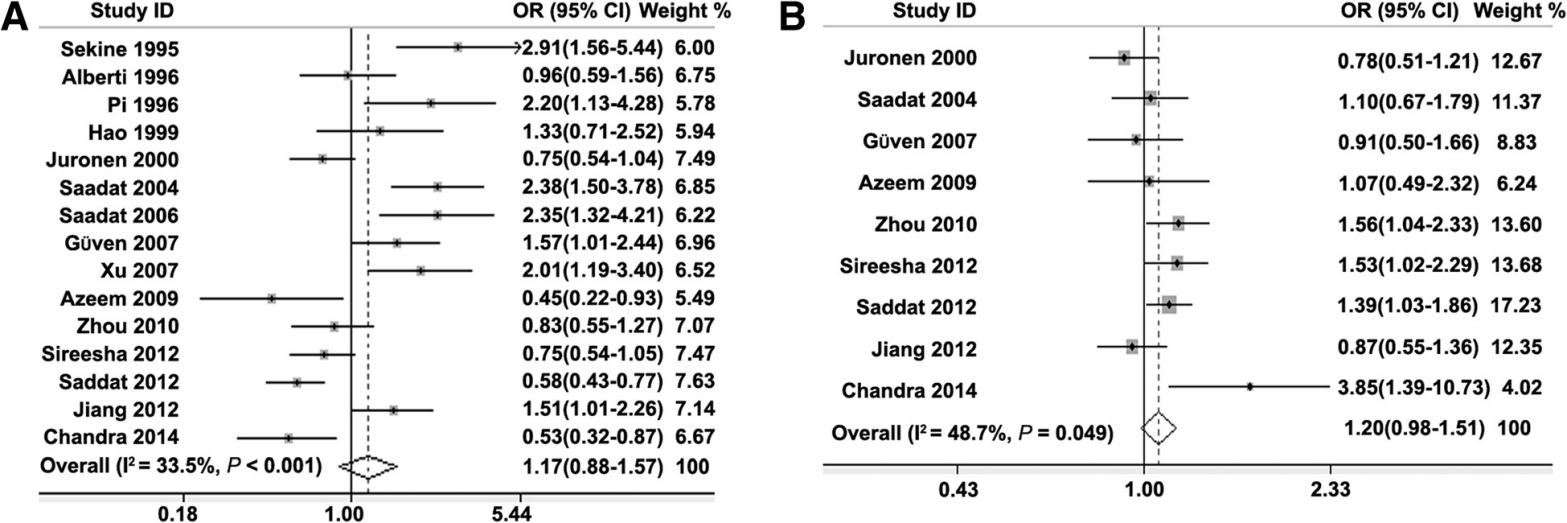
Forest plots of the association between *GSTM1* or *GSTT1* null polymorphism and cataract risk. The random-effects or fixed model was used to calculate the pooled effect estimates of the effects of *GSTM1* (a) or *GSTT1* (b) null polymorphism on cataract risk respectively. The squares and horizontal lines correspond to OR and 95 % CI of specific study, and the area of squares reflects study weight (inverse of the variance). The diamond represents the pooled OR and its 95 % CI

Table 2 shows the results of the meta-analysis on the association of *GSTM1* or *GSTT1* null polymorphism with cataract risk. When pooling all the studies, we found that *GSTM1* null polymorphism was not associated with cataract risk (Fig. 2a), and this negative association maintained in either Caucasian or Asian populations (Table 2). When stratified by the source of controls, gender, or cataract subtype, no association was found between *GSTM1* null polymorphism and cataract risk.

**Table 2:** Association between *GSTM1* or *GSTT1* polymorphism and cataract risk

Groups	N^a^	Statistical method^b^	OR (95 % CI)	*P*
*GSTM1*				
All	15	Random (*P* < 0.001)	1.17 (0.88–1.57)	0.288
Ethnics				
Caucasian	9	Random (*P* < 0.001)	1.07 (0.753–1.53)	0.712
Asian	6	Random (*P* < 0.001)	1.37 (0.79–2.40)	0.266
Study design				
Population-based	3	Random (*P* = 0.001)	1.17 (0.58–2.33)	0.666
Hospital-based	12	Random (*P* < 0.001)	1.18 (0.84–1.65)	0.350
Gender				
Male	5	Random (*P* = 0.035)	0.89 (0.58–1.37)	0.598
Female	5	Random (*P* < 0.001)	1.02 (0.44–2.32)	0.970
Subtype				
Cortical	4	Random (*P* = 0.086)	0.85 (0.59–1.23)	0.386
Nuclear	4	Random (*P* = 0.084)	0.97 (0.62–1.52)	0.904
Posterior subcapsular	3	Fixed (*P* = 0.242)	0.98 (0.72–1.32)	0.879
Mixed	4	Random (*P* = 0.040)	0.94 (0.60–1.48)	0.792
*GSTT1*				
All	9	Random (*P* = 0.049)	1.20 (0.96–1.51)	0.105
Ethnics				
Caucasian	7	Random (*P* = 0.058)	1.11 (0.83–1.49)	0.474
Asian	2	Fixed (*P* = 0.653)	1.44 (1.14–1.83)	0.003
Study design				
Population-based	2	Fixed (*P* = 0.952)	1.54 (1.16–2.05)	0.003
Hospital-based	7	Random (*P* = 0.063)	1.10 (0.84–1.45)	0.498
Gender				
Male	5	Fixed (*P* = 0.984)	1.29 (0.98–1.70)	0.073
Female	5	Fixed (*P* = 0.359)	1.28 (0.97–1.69)	0.078
Subtype				
Cortical	4	Fixed (*P* = 0.186)	1.09 (0.82–1.45)	0.555
Nuclear	4	Random (*P* = 0.062)	0.92 (0.52–1.62)	0.774
Posterior subcapsular	4	Fixed (*P* = 0.219)	1.42 (1.04–1.94)	0.026
Mixed	3	Random (*P* = 0.097)	1.21 (0.66–2.20)	0.535

^a^N: The number of included studies

^b^A random-effects or fixed-effects model was used in presence (*P* ≤ 0.10) or absence (*P* > 0.10) of heterogeneity of included studies and the *P* value was presented in parenthesis

For *GSTT1*, the overall result showed that *GSTT1* null polymorphism was significantly associated with increased risk of cataract in Asian (OR, 1.44; 95 % CI, 1.14–1.83) but not Caucasian populations (Table 2). The positive association of *GSTT1* null polymorphism with increased risk of cataract was found when pooling studies with population-based (OR, 1.54; 95 % CI, 1.16–2.05) but not hospital-based controls. However, there was no association between *GSTT1* null polymorphism and cataract risk in male or female subjects. Interestingly, *GSTT1* null polymorphism was associated with risk of posterior subcapsular (OR, 1.42; 95 % CI, 1.04–1.94) but not other subtypes of cataract.

We next investigated the effects of the profiles of *GST* genotypes on the risk of cataract, and examined the association between combinations of *GSTM1* and *GSTT1* null genotypes and cataract risk. Table 3 displays cataract risk associated with combinations of *GST* null genotypes, and the trend in risk associated with each putative high-risk null genotype. The results showed no association between the combined *GSTM1* and *GSTT1* null genotypes and cataract risk in all population, Caucasian or Asian population. When stratified by source of controls, pooled two studies with population-based controls showed that combination of *GSTM1* null and *GSTT1* positive (*GSTT1*^*+/+*^) genotypes played a protective role in cataract risk (OR, 0.71; 95 % CI, 0.54–0.92), but combination of *GSTM1* positive and *GSTT1* null, or *GSTM1* and *GSTT1* null genotypes was not associated with cataract risk. The other sub-group analyses showed no association between combination of *GSTM1* and *GSTT1* polymorphisms and cataract risk.

**Table 3:** Association between *GSTM1* and *GSTT1* polymorphisms and cataract risk

Groups	Number^a^	Statistical method^b^	OR (95 % CI)	*P*
All				
*GSTM1* null + *GSTT1* positive	7	Random (*P* < 0.001)	0.83 (0.56– 1.23)	0.356
*GSTM1* positive + *GSTT1* null	7	Fixed (*P* = 0.240)	1.20 (0.95– 1.53)	0.134
*GSTM1* null + *GSTT1* null	7	Random (*P* = 0.010)	1.16 (0.71– 1.89)	0.545
Ethnics				
Caucasian				
*GSTM1* null + *GSTT1* positive	6	Random (*P* < 0.001)	0.85 (0.52– 1.37)	0.494
*GSTM1* positive + *GSTT1* null	6	Fixed (*P* = 0.658)	1.00 (0.74– 1.34)	0.983
*GSTM1* null + *GSTT1* null	6	Random (*P* = 0.008)	1.27 (0.67– 2.38)	0.466
Study design				
PB				
*GSTM1* null + *GSTT1* positive	2	Fixed (*P* = 0.591)	0.71 (0.54– 0.92)	0.009
*GSTM1* positive + *GSTT1* null	2	Fixed (*P* = 0.334)	1.03 (0.69– 1.53)	0.899
*GSTM1* null + *GSTT1* null	2	Random (*P* = 0.036)	0.87 (0.34– 2.18)	0.760
HB				
*GSTM1* null + *GSTT1* positive	5	Random (*P* < 0.001)	0.88 (0.47– 1.65)	0.697
*GSTM1* positive + *GSTT1* null	5	Fixed (*P* = 0.196)	1.32 (0.97– 1.79)	0.073
*GSTM1* null + *GSTT1* null	5	Random (*P* = 0.024)	1.38 (0.71– 2.69)	0.336
Gender				
Male				
*GSTM1* null + *GSTT1* positive	2	Fixed (*P* = 0.990)	0.88 (0.49– 1.59)	0.676
*GSTM1* positive + *GSTT1* null	2	Fixed (*P* = 0.476)	0.84 (0.28– 2.50)	0.749
*GSTM1* null + *GSTT1* null	2	Fixed (*P* = 0.672)	1.48 (0.52– 4.21)	0.463
Female				
*GSTM1* null + *GSTT1* positive	2	Random (*P* < 0.001)	0.79 (0.06– 10.87)	0.858
*GSTM1* positive + *GSTT1* null	2	Fixed (*P* = 0.767)	0.62 (0.27– 1.43)	0.264
*GSTM1* null + *GSTT1* null	2	Random (*P* = 0.074)	0.91 (0.15– 5.57)	0.919
Cataract type				
Cortical				
*GSTM1* null + *GSTT1* positive	3	Fixed (*P* = 0.745)	0.82 (0.62– 1.10)	0.181
*GSTM1* positive + *GSTT1* null	3	Fixed (*P* = 0.131)	1.39 (0.99– 1.96)	0.061
*GSTM1* null + *GSTT1* null	3	Fixed (*P* = 0.171)	1.03 (0.72– 1.48)	0.855
Nuclear				
*GSTM1* null + *GSTT1* positive	2	Random (*P* = 0.030)	1.00 (0.39– 2.56)	0.994
*GSTM1* positive + *GSTT1* null	2	Random (*P* = 0.081)	0.67 (0.11– 4.24)	0.668
*GSTM1* null + *GSTT1* null	2	Fixed (*P* = 0.868)	1.16 (0.56– 2.38)	0.694
Posterior subcapsular				
*GSTM1* null + *GSTT1* positive	2	Random (*P* = 0.038)	1.20 (0.42– 3.39)	0.734
*GSTM1* positive + *GSTT1* null	2	Fixed (*P* = 0.157)	1.15 (0.59– 2.26)	0.682
*GSTM1* null + *GSTT1* null	2	Fixed (*P* = 0.399)	1.97 (0.98– 3.97)	0.059
Mixed				
*GSTM1* null + *GSTT1* positive	2	Random (*P* = 0.019)	0.81 (0.25– 2.61)	0.724
*GSTM1* positive + *GSTT1* null	2	Fixed (*P* = 0.130)	1.22 (0.68– 2.21)	0.505
*GSTM1* null + *GSTT1* null	2	Fixed (*P* = 0.523)	1.44 (0.74– 2.79)	0.279

^a^N: The number of included studies

^b^A random-effects or fixed-effects model was used in presence (*P* ≤ 0.10) or absence (*P* > 0.10) of heterogeneity of included studies and the *P* value was presented in parenthesis

### Potential publication bias and sensitivity analysis

**Fig. 3:**
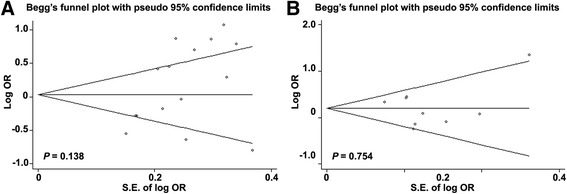
Funnel plots showed symmetric distribution. Log OR is plotted against the standard error of log OR for studies on *GSTM1* (a) or *GSTT1* null (b) polymorphism. The dots represent specific studies for the indicated association

We firstly detected the publication bias by the Begg’s test for the overall pooled analyses of *GSTM1* and *GSTT1* null genotype, and found symmetric distribution of corresponding funnel plots for *GSTM1* genotype with a *P* value of 0.138, and *GSTT1* genotype with a *P* value of 0.754 (Fig. 3). However, the Egger’s test showed that the *P* values for *GSTM1* and *GSTT1* null genotype were 0.037 and 0.908 respectively, suggesting a publication bias for studies on *GSTM1* but not *GSTT1* genotype.

**Fig. 4:**
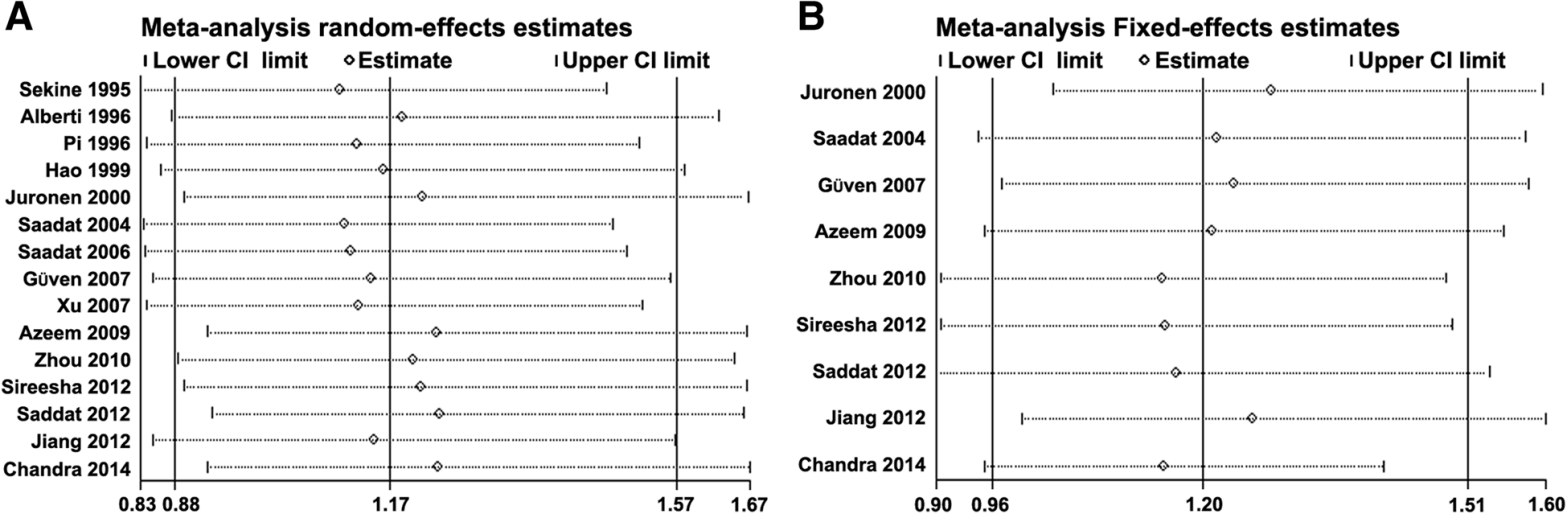
Sensitivity analyses for *GSTM1* or *GSTT1* null polymorphism. Sensitivity analysis was performed for *GSTM1* (a) or *GSTT1* null (b) polymorphism. Each study was deleted at a time in synthetic analysis to detect the influence of the omitted study. The hollow circles represent OR of pooled results with the deletion of each study. The ranges of horizontal dotted-lines represent the 95 % confidence intervals of the corresponding OR

Sensitivity analysis showed that exclusion of each study did not influence the result in specific genotype comparison for *GSTM1* and *GSTT1* polymorphism (Fig. 4), suggesting that the results of synthetic analysis were robust.

## Discussion

Before inclusion of studies, we briefly searched PubMed, Embase, Web of Science and China National Knowledge Infrastructure, and found that most of studies examined association of *GSTM1* or *GSTT1* polymorphisms with cataract risk while very limited studies were related to other *GST* polymorphisms, e.g., *GSTM3*, *GSTO* or *GSTP* polymorphisms. Thus, this meta-analysis only evaluated the effects of *GSTM1* and *GSTT1* ploymorphisms on cataract risk. Our data showed that *GSTT1* but not *GSTM1* null polymorphism was associated with cataract risk in Asians. Although different subtypes of cataract have their own pathogenesis and clinical characteristics, our meta-analysis data indicate that *GSTT1* null polymorphism may contribute to increased risk of posterior subcapsular cataract.

In 1995, Sekine and colleagues for the first time reported possible correlation of *GSTM1* null genotype frequency with cataract risk [36]. However, the following studies showed inconsistent results [18, 21–25, 32–35]. By pooling these early studies, previous meta-analysis by Sun *et al*., did not find an association of *GSTM1* null genotype with cataract risk [6]. Even including three more studies, we did not find positive relationship between *GSTM1* null genotype and cataract risk. To be noted, although previous meta-analysis indicated an association of *GSTM1* null genotype and increased risk of cataract in Asians [6], our data did not confirm this association when including one more study on Asians.

For *GSTT1* polymorphism, pooled four early studies on Caucasian showed no association [18, 22, 24, 25] while one study on Asians [21] showed positive association between *GSTT1* null genotype and cataract risk; however, by pooling these five studies, no association was found [6]. By including four recent studies, our meta-analysis showed positive association of *GSTT1* polymorphism with increased risk of cataract in all populations, and this association remained in Asians when two studies were pooling [15, 21]. Previous studies reported gender-dependent effects of *GSTT1* null polymorphism on cataract risk [18, 22, 24]; however, recent two studies showed negative results [15, 16]. We performed a subgroup analysis stratified by gender with all five studies, and results showed no significant association, which was consistent with previous meta-analysis data based on three studies [6]. In addition, our data showed positive association of *GSTT1* null polymorphism with increased risk of posterior subcapsular cataract although previous pooled study indicated that this association did not reach significant (OR, 1.21; 95 % CI, 0.96–1.53) [6]. Since the studies included for subgroup analyses were still limited, future studies are required to validate the association between *GSTT1* null polymorphism and cataract risk.

To the best of our knowledge, the association between combination of *GST* polymorphisms and susceptibility to cataract has been assessed for the first time by our meta-analysis. The study by Juronen *et al*., firstly reported that the *GSTM1* positive phenotype frequency was significantly higher in the cataract group than in the controls, and the cataract risk associated with the *GSTM1* positive phenotype was increased in carriers of the combined *GSTM1* positive and *GSTT1* positive phenotypes [25]. However, a later study by Saadat *et al*., showed that individuals with the null genotypes for *GSTM1* and *GSTT1*, or combination of *GSTT1* positive and *GSTM1* null genotypes were at a significantly higher risk for developing cataract than individuals with both the genes positive genotypes [24]. The following studies consecutively presented inconsistent results [15, 16, 18, 22]. By pooling seven studies, our meta-analysis results did not show a significant association between each combination of *GSTM1* and *GSTT1* genotypes and cataract risk. Two pooled studies with population-based controls showed that combination of *GSTM1* null and *GSTT1* positive genotypes played a protective role in cataract risk [16, 25]; however, this positive association was not found in other stratified analyses. Thus, the result should be interpreted with caution.

When compared to individual studies, the meta-analysis has a vital advantages. However, some potential limitations in our study should be considered. First, the inclusion of studies might not be sufficient since we only included published papers with language in English, or Chinese. It is possible that some papers published in other languages may not indexed by the database (e.g. PubMed, Embase, Web of Science). Thus, the publication bias for *GSTM1* polymorphism detected in our study might be due to insufficient inclusion of published studies. Second, this meta-analysis was limited by the small sample size, especially in subgroup analyses aforementioned (e.g., studies on *GSTT1* polymorphism in Asians), and this need further investigation. Third, basic methodological differences among the studies, e.g., ELISA *v*s. PCR assay for genotyping, might have affected the results. Fourth, most of the studies included did not categorize the cataract patients as cortical, nuclear, posterior subcapsular and mixed cataract. Although we found positive association between *GSTT1* null polymorphism and increased risk of posterior subcapsular cataract, however, only four studies with available data were pooled [16, 18, 21, 25], and thus this association awaits further confirmation. Fifth, the primary outcome measure was calculated based on individual unadjusted ORs, which might affect the evaluation precision of the study. The lack of detailed data in each study prevented multiple testing for combined effects of gene-environment factors on cataract risk, and thus future studies should address this point. Last, the Caucasian and Asian subjects from different countries might have been genetically heterogeneous, e.g., different lifestyle and environment (e.g., European *vs*. Arabian). These factors may explain the heterogeneity in this meta-analysis for Caucasian subjects.

## Conclusion

In summary, the present meta-analysis showed that the association between *GSTM1* null polymorphism and cataract risk was either negative or evidence limited. The *GSTT1* null polymorphism was significantly associated with increased risk of posterior subcapsular cataract. Given the limited study populations, more studies with large study population are suggested to further validate the relationship between *GST* polymorphisms and genetic predisposition to cataract, e.g., association of *GSTT1* null polymorphism with cataract risk in Asian.
